# HLA-B*1301 as a Biomarker for Genetic Susceptibility to Hypersensitivity Dermatitis Induced by Trichloroethylene among Workers in China

**DOI:** 10.1289/ehp.10325

**Published:** 2007-08-24

**Authors:** Haishan Li, Yufei Dai, Hanlin Huang, Laiyu Li, Shuguang Leng, Juan Cheng, Yong Niu, Huawei Duan, Qingjun Liu, Xing Zhang, Xianqing Huang, Jinxin Xie, Zhiming Feng, Juncai Wang, Jiaxi He, Yuxin Zheng

**Affiliations:** 1 Key Laboratory, National Institute for Occupational Health and Poison Control, Chinese Center for Disease Control and Prevention, Beijing, China; 2 Department of Toxicology, Hospital for Occupational Diseases Control of Guangdong Province, Guangzhou, Guangdong, China; 3 Hospital for Occupational Diseases Control of Shenzhen, Shenzhen, Guangdong, China; 4 Institute of Health Inspection of Longgang District, Shenzhen, Guangdong, China

**Keywords:** biomarkers, genetic polymorphism, human leukocyte antigen, hypersensitivity dermatitis, trichloroethylene

## Abstract

**Background:**

Trichloroethylene (TCE) is used extensively as an industrial solvent and has been recognized as one of the major environmental pollutants. To date, > 200 cases of TCE-induced hypersensitivity dermatitis among exposed workers have been reported worldwide, and TCE exposure has become one of the critical occupational health issues in Asia.

**Objectives:**

The study aimed to identify genetic susceptible biomarkers associated with the TCE-induced hypersensitivity dermatitis in genes located in the human leukocyte antigen (HLA) region.

**Methods:**

From 1998 to 2006, 121 cases with TCE-induced hypersensitivity dermatitis and 142 tolerant controls were recruited into the population-based case–control study. We determined *HLA* alleles *B*, *DRB1*, *DQA1,* and *DQB1*, by sequence-based typing. *p*-Values were corrected for comparisons of multiple *HLA* alleles. In addition, we compared and analyzed the structure character of amino acid residues of HLA molecules found in participants.

**Results:**

We obtained complete genotyping data of 113 cases and 142 controls. The allele HLA-B*1301 was present in 83 (73.5%) of 113 patients compared with 13 (9.2%) of 142 tolerant workers (odds ratio = 27.5; 95% confidence interval, 13.5–55.7; corrected *p* = 1.48 × 10^−21^). In addition, the HLA-B*44 alleles were present in 6.2% (7/113) of patients, but were absent in TCE-tolerant workers. Residue 95 shared by HLA-B*1301 and HLA-B*44 molecules formed a different pocket F than other residues.

**Conclusions:**

The allele HLA-B*1301 is strongly associated with TCE-induced hypersensitivity dermatitis among exposed workers and might be used as a biomarker to predict high risk individuals to TCE.

Trichloroethylene (TCE) is known as a major pollutant that affects both the occupational and general environment. It has been used as an industrial degreasing agent, solvent, and extraction agent for approximately a century. Alternatively, the general population may be exposed to TCE through contaminated drinking water or air. The target organs of TCE include the nervous system, 1iver, kidney, heart, and skin (Bruckner et al. 1989). Among the immunotoxicity end points, evidence for an effect of TCE was strongest for auto-immune disease. Studies in susceptible rodents have shown that TCE exacerbates underlying autoimmune disease, and supporting information comes from multiple human studies of scleroderma and exposure to organic solvents (National Research Council 2006).

Hypersensitivity dermatitis occurring in workers exposed to TCE is completely different from nonspecific skin irritation as a result of defatting action, and the serious consequences of TCE exposure have recently become one of the critical occupational health issues in Asia. The clinical manifestations of TCE exposure include generalized severe dermatitis, fever, abnormal liver function, jaundice, and lymphadenopathy. Skin lesions range from mild multiform erythema to lesions with increasing severity, exfoliative dermatitis, Stevens-Johnson syndrome, and toxic epidermal necrolysis (Huang et al. 2006). The literature published in English and ad hoc publications in local languages were reviewed in detail recently by Kamijima et al. (2007). Occurrences of the disease have been reported from the United States, Japan, Spain, Singapore, China, Korea, Thailand, and the Philippines. Most case reports from industrialized countries were published up to 1990, whereas cases from Asian industrializing countries were published later. In the past 10 years, an increasing number of cases of hypersensitivity dermatitis have been reported among TCE-exposed workers from electronic-element plants in Guangdong Province in southern China (Huang et al. 2002; Kamijima et al. 2007).

According to epidemiologic surveys in Guangdong Province, > 200 cases of hypersensitivity dermatitis have been diagnosed, with a prevalence rate of < 1% among TCE-exposed workers (Huang and Li 2006). Epidemiologic studies on patients with TCE-induced hypersensitivity dermatitis reveal that the latency ranges from 6 to 89 days, and a no dose–response relationship was observed (Li et al. 2006). Furthermore, the patch test using TCE and/or its oxidative metabolites among victims was positive (Huang et al. 2002).

The incidence of hypersensitivity and autoimmune diseases in industrialized countries has steadily increased over the past three decades (Bach 2002), and these diseases are not evenly distributed among countries, regions, or ethnic groups. However, the degree to which genetic and environmental factors influence susceptibility to hypersensitivity and autoimmune diseases is not well defined. One main feature of occupational diseases is relatively defined causative factors, so studies on these diseases are helpful in identifying the role of genetic factors.

One of the best ways to prevent occupational hypersensitivity disease is to identify susceptible biomarkers to use in screening employees before exposure. To date, however, there are no available biomarkers for predicting who among TCE-exposed workers is at high risk of developing hypersensitivity dermatitis. Other factors associated with the disease remain unknown except for human herpes virus 6 reactivation (Huang et al. 2006). The genomic region of human leukocyte antigen (HLA) at chromosomal position 6p21 encodes the six classical HLA genes and many other genes that have important roles in the regulation of the immune system as well as in some fundamental cellular processes. The classical class I (HLA-A, HLA-B, and HLA-C) and class II molecules (HLA-DR, HLA-DQ, and HLA-DP) are involved in the control of immune-response mediation of antigen presentation to the T lymphocytes, and play a central role in the immune system by presenting peptides (Shiina et al. 2004). The HLAs are characterized by an extensive degree of allelic polymorphism; strong associations between hypersensitivity or autoimmune disease and certain *HLA* alleles have been reported, for instance, HLA-B*27 and ankylosing spondylitis (Brewerton et al. 1973), and HLA-B*1502 and carbamazepine-induced Stevens-Johnson syndrome (Chung et al. 2004). Therefore, we hypothesized that the polymorphisms of *HLA* loci might influence the susceptibility of TCE-induced hypersensitivity dermatitis. We conducted a population-based case–control study to investigate this hypothesis.

## Materials and Methods

### Study population

From June 1998 to March 2006, 121 cases with TCE-induced hypersensitivity dermatitis and 142 TCE-tolerant controls were recruited into the case–control study. Both cases and controls were recruited from 80 factories engaged in electronic-element and metal-plating production in Guangdong Province, China. At least one patient was identified in each factory. Controls were defined as the co-workers of the patients with same job title and longer occupational exposure time (> 90 days) but no skin abnormalities detected by the occupational physicians upon examination. According to data of routine environmental monitoring in the years the cases occurred, the TCE levels in these workplaces ranged from 69 to 790 ppm (median concentration, 90 ppm), which is much higher than the permissible concentration time-weighted average of 5.6 ppm defined by the Ministry of Health, China (2002b). Compared with the exposure data reviewed by Kamijima et al. (2007), the environmental exposure concentrations in the present study were relatively high. All participants were from the southern region of China and were genetically unrelated. Both cases and controls were exposed to TCE in the workplace where they cleaned and degreased metals. The cases were diagnosed with occupational hypersensitivity dermatitis by a panel of occupational physicians according to Chinese National Diagnostic Criteria of Occupational Disease (GBZ38–2002) (2002a). Briefly, on the basis of both TCE exposure history and clinical manifestations (in terms of dermatitis accompanied by fever, lymphadenopathy, and hepatitis), patients were judged to be cases if the onset of symptoms occurred within the first 3 months of TCE exposure. Other potential risk factors, such as medications used and the history of previous skin disease, were excluded during diagnosis.

Four clinical categories were defined among the cases. First, exfoliative dermatitis is characterized by cutaneous fine pink macules and multiorgan involvement (e.g., hepatitis, nephritis) accompanied by systemic manifestations (e.g., fever, eosinophilia, lymphadenopathy, hepatomegaly, splenomegaly) in addition to skin rashes. Second, multiform erythema is characterized by different forms of cutaneous lesions, such as exanthema, blisters, and erythema exsudativum, without mucosal involvement. Third, Stevens-Johnson syndrome is characterized by a rapidly developing blistering exanthema of purpuric macules and target-like lesions accompanied by mucosal involvement. Fourth, toxic epidermal necrolysis is characterized by skin detachment of > 30% of body surface area. The diagnosis of each case was confirmed by occupational panels. The clinical classification of the disease has been described in detail by Kamijima et al. (2007).

The study was approved by the local institutional review board, and informed consent was obtained from all participants.

### HLA genotyping

We obtained genomic DNA from blood by the high-salt method. HLA alleles *HLA-B* [UniGene accession no. Hs.77961 (Unigene 2007)], *HLA-DRB1* (UniGene accession no. Hs.654405), *HLA-DQA1* (UniGene accession no. Hs.387679), and *HLA-DQB1* (UniGene accession no. Hs.409934), were determined by sequence-based typing protocols (Luo et al. 1999; Pozzi et al. 1999; Sayer et al. 2004). We then performed bidirectional sequencing. In brief, two reactions were used to obtain sequences of both DNA strands of exon 2 for *HLA-DQA1*, *DQB1*, and *DRB1*, and four reactions were used to obtain sequences of both DNA strands of exon 2 and exon 3 for *HLA-B*. We confirmed ambiguity in equivocal combination results by clone sequencing. We obtained complete sequencing data for 113 cases and 142 controls; because of insufficient DNA quality or quantity, DNA samples from 8 cases were excluded from sequencing.

### Comparison of amino acid residues

We translated the amino acid sequences of 1–182 codons (peptide-binding regions) of HLA*B alleles found in all participants according to the DNA sequence, and compared the amino acid residues of HLA molecules that were positively or nonpositively associated with hypersensitivity disease. We downloaded three-dimensional (3D) HLA homology models from the FIMM website (FIMM 2003); the structural models were generated by D.R. Flower (Edward Jenner Institute for Vaccine Research, Compton, Berkshire, UK).

### Statistical analysis

We compared genotype frequencies between groups using Fisher’s exact test. To adjust for multiple comparisons, we calculated corrected *p* (*p*_c_) values using Bonferroni’s correction for all observed alleles (68 for *HLA-B*, 8 for *HLA-DQA1*, 28 for *HLA-DQB1*, and 78 for *HLA-DRB1*). Therefore, the *p*_c_ value is equal to the *p*-value multiplied by a factor of 1,188,096. Because age and sex were well matched between cases and controls (Table 1), the odds ratios (ORs) and their 95% confidence intervals (CIs) were calculated to estimate the relative risk. To accommodate zero count, ORs were calculated with Haldane’s modification, which adds the value of 0.5 to each of the cells with zero observation. Hierarchical cluster analysis was used for comparison of amino acid residues encoded by *HLA-B* alleles.

## Results

### Characteristics of cases and controls

The patients typically showed a rash on the extremities, face, neck, or trunk with or without fever within 14–40 days after occupational TCE exposure commenced. The age, sex, exposure duration, and major clinical manifestations are summarized in Table 1. Exfoliative dermatitis was the most common clinical manifestation (68.1%; 77/113). We found six cases with Stevens-Johnson syndrome and five cases with toxic epidermal necrolysis; only one case was diagnosed with Stevens-Johnson syndrome in combination with toxic epidermal necrolysis. Because Stevens-Johnson syndrome and toxic epidermal necrolysis are considered to be the same disease but with varying degrees of severity, we grouped them together for further analysis (Kamijima et al. 2007). As shown in Table 1, there was no significant difference in distributions of sex and age among these subgroups.

### HLA allele frequency

Notably, we found that the allele HLA-B*1301 was present in 73.5% (83/113) of TCE-induced hypersensitivity dermatitis patients but only in 9.2% (13/142) of TCE-tolerant workers (OR = 27.5; 95% CI, 13.5–55.7; *p*_c_ = 1.48 × 10^−21^). In addition, the HLA-B*44 alleles, including B*4402 and B*4403, were present in 6.2% (7/113) of cases, whereas in TCE-tolerant workers the alleles were absent (Table 2). In contrast, we found no significant association between *HLA-DRB1*, *HLA-DQA1*, or *HLA-DQB1* polymorphisms and the occurrence of TCE-induced hypersensitivity dermatitis (data not shown).

By taking clinical categories into consideration, we found no significant difference in frequencies of HLA-B*1301 among the three subgroups (data not shown).

### Characteristics of amino acid residues

**Figure 1:**
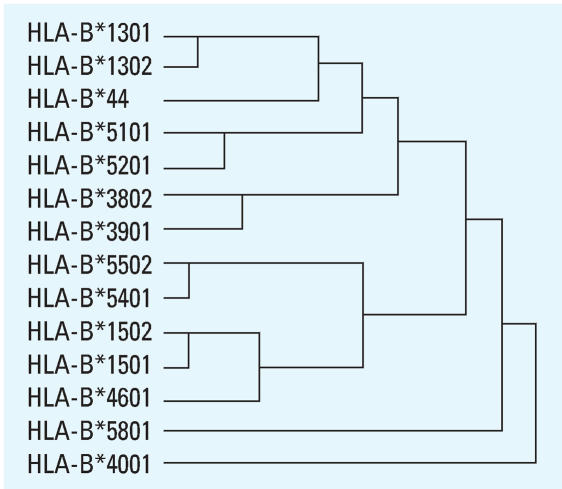
Hierarchical cluster of 1–182 amino acid residues of HLA-B alleles were positive and non-positive associated with trichloroethylene-induced hypersensitivity dermatitis. Alleles that phenotype frequencies below 3% in controls were not involved.

Comparison of 1–182 codons (peptide-binding regions) of 68 HLA*B alleles in all participants showed that HLA-B*1301 and HLA-B*44 shared 171 amino acid residues. Hierarchical cluster analysis also showed that HLA-B*13 and HLA-B*44 have similar amino acid residues (Figure 1).

**Figure 2:**
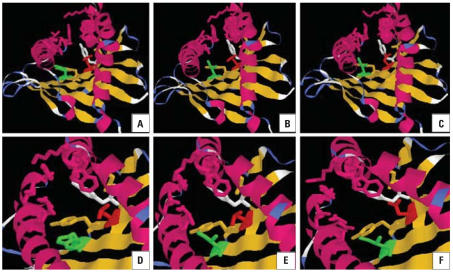
Comparison of pocket F in tertiary structures among HLA-B*1302 (*A*), HLA-B*1301 (*B*), and HLA-B*44 (*C*). The corresponding magnified images (*D, E,* and *F,* respectively) emphasize the conformational changes in residue 95 (green) and residue 116 (red).

Although only three residues were different between HLA-B*1301 and HLA-B*1302, only HLA-B*1301 was related to TCE-induced hypersensitivity dermatitis. The different residues were I^94^I^95^R^97^ (HLA-B*1301) versus T^94^W^95^T^97^ (HLA-B*1302). Residue 95 was a component of peptide-binding pocket F. Amino acid residues 73, 77, 80, 81, 84, 95, 116, 118, 123, 124, 143, 146, and 147 constitute pocket F of HLA class I molecules (FIMM 2003). One different residue was found between HLA-B*1301 and HLA-B*1302 (I^95^ vs. W^95^). Between HLA-B*1301 and HLA-B*44, there was a difference in residue 116 (L^116^ vs. D^116^). Observations based on HLA 3D homology models showed that residue 95, the bottom of the pocket F, formed a flat structure in W^95^ (tryptophan) but not in I^95^ (isoleucine). In contrast, a similar structure is present between L^116^ and D^116^ (Figure 2).

## Discussion

HLA-B*13 is one of a common allelic group (phenotype frequency ranges from 10 to 30%) found in the Chinese population, and the distribution of *HLA-B* alleles in the controls of the present study was similar to that of the general population in southern China (Feng et al. 2004; Liang et al. 2005; Shi et al. 2006; Yang et al. 2006). In addition, the HLA-B*1301 allele, a member of the HLA-B*13 group in the southern Chinese population, is a specific allele in Asians, but is absent in whites and blacks (Cao et al. 2001; Lin et al. 1995; Williams et al. 2001). In contrast, HLA-B*1302 is the predominant allele of the HLA-B*13 group in whites (Cao et al. 2001; Williams et al. 2001). The frequencies of HLA-B*1301 and HLA-B*1302 are variably distributed in different regions of China. The frequency of HLA-B*1301 in southern Chinese is higher than that in northern Chinese (Feng et al. 2004; Middleton et al. 2004; Shi et al. 2006; Yang et al. 2006). Rapid economic development, as occurring in China, could generate a highly mobile work force. As such, large numbers of new TCE-exposed workers, combined with genetic features of the population in southern China, may be a major factor in the mass outbreaks of TCE-induced hypersensitivity dermatitis.

The peptides that bind to HLA class I molecules are restricted in length and often contain key amino acids (anchor residues) at certain positions. The side-chains of the peptide anchor residues interact with the polymorphic complementary pockets in HLA peptide-binding grooves and provide the molecular basis for allele-specific recognition of antigenic peptides. HLA polymorphic side-chains delineate the geometry and chemical properties of six structural pockets (A–F) in the groove that can accommodate some of the peptide side-chains. Specifically, the C-terminal side-chain of the peptide, which is lodged in pocket F close to one end of the groove, is important to the stability of the HLA/peptide complex (Rammensee et al. 1995). Comparison of amino acid residues between HLA-B*1301 and HLA-B*1302 showed that only three residues (I^94^I^95^R^97^ compared with T^94^W^95^T^97^) were different, and residue 95 is a major component of pocket F. By generating recombinant HLA-B molecules and studying the eluted peptides by mass spectrometry and pool sequencing, Bade-Doeding et al. (2007) found that residue 95 causes strong alteration of peptide motifs. According to structure–function analysis, residue I^95^ might play a key role in susceptibility to TCE-induced hypersensitivity dermatitis.

In addition, HLA-B*44 was present in 6.2% of cases but was not observed in any of the 142 TCE-tolerant workers. Noticeably, HLA-B*1301 and HLA-B*44 shared I^94^I^95^R^97^, which might play a key role in susceptibility to TCE-induced hypersensitivity dermatitis described above. In a previous study that included 15 white patients, Mondino et al. (1982) found that HLA-B*44 was associated with the risk of Stevens-Johnson syndrome with ocular involvement; therefore, it is possible that the weak association of HLA-B*44 with hypersensitivity dermatitis in TCE-exposed workers is not a chance finding. Certainly, this association should be cautiously considered because of the small number of patients with the HLA-B*44 allele.

Recently, strong associations have been reported between a particular *HLA* allele and hypersensitivity dermatitis in patients under treatment with several drugs. Hypersensitivity to abacavir, a nucleoside reverse-transcriptase inhibitor commonly used for anti-AIDS treatment, has been reported to be associated with an *HLA-B* allele (HLA-B*5701) and a haplo-typic *Hsp70-Hom* variant in the HLA region (Mallal et al. 2002; Martin et al. 2004). Chung et al. (2004) reported a strong association of a specific *HLA-B* allele (HLA-B*1502) in Chinese patients with carbamazepine-induced Stevens-Johnson syndrome. In addition, a significant association was reported between another *HLA-B* allele (HLA-B*5801) and allopurinol-induced cutaneous adverse reactions both in Chinese patients and in European patients (Hung et al. 2005; Lonjou et al. 2006). Interestingly, HLA-B*1301, HLA-B*1502, and HLA-B*5801, three common alleles in southern Chinese, shared the residues I^94^I^95^R^97^, which differed from almost all other residues present in all participants. In contrast, HLA-B*1302 and HLA-B*1501 did not share these residues. Therefore, data from these studies and from the present study suggest that there is a common molecular basis for using HLA-B*1301, HLA-B*1502, and HLA-B*5801 as genetic markers for chemical hypersensitivity dermatitis in the Chinese population.

Taken together, these studies suggest that *HLA-B* alleles—alone or together with other genetic polymorphisms in the HLA region—might play a pivotal role in determining individual susceptibility to hypersensitivity dermatitis induced by certain chemicals. Therefore, the mechanism of *HLA-B* involved in severe hypersensitivity dermatitis requires further investigation along these lines.

To our knowledge, this is the first report of the association of the HLA-B*1301 allele with human disease, and it is the strongest association between an *HLA* marker and a chemical-related occupational disease described thus far. Potentially, this association could be used to develop a highly reliable screening test to predict high-risk subpopulations to TCE exposure and could be applied for occupational practice to prevent about 70% of the cases of this life-threatening disease. In summary, HLA-B*1301 and HLA-B*44 might play a crucial role in the pathogenesis of TCE-induced hypersensitivity dermatitis.

## Figures and Tables

**Table 1:** Demographic data for the cases and controls.

	Cases				
Variable	ED (*n* = 77)	ME (*n* = 25)	SJS/TEN (*n* = 11)	Total (*n* = 113)	Controls (*n* = 142)
Age [years (mean ± SD)]	22.9 ± 5.41	22.0 ± 4.18	23.7 ± 6.71	22.8 ± 5.28	23.4 ± 5.24
Male [no. (%)]	32 (41.6)	15 (60.0)	6 (54.5)	53 (46.9)	67 (47.2)
Female [no. (%)]	45 (58.4)	10 (40.0)	5 (45.5)	60 (53.1)	75 (52.8)
TCE exposure duration (months)	< 3	< 3	< 3	< 3	> 3
Lymphadenopathy [no. (%)]	56 (72.7)	17 (68.0)	10 (90.9)	83 (73.5)	0 (0.0)
Hepatitis [no. (%)]	64 (83.1)	20 (80.0)	11 (100.0)	95 (84.0)	0 (0.0)

Abbreviations: ED, exfoliative dermatitis; ME, multiform erythema; SJS, Stevens-Johnson syndrome; TEN, toxic epidermal necrolysis.

**Table 2:** Frequencies of *HLA-B* alleles in cases and controls.

HLA allele	Cases (*n* = 113)	Controls (*n* = 142)	OR (95% CI)	*p-*Value	*p*_c_
B*1301	83 (73.5%)	13 (9.2%)	27.5 (13.5–55.7)	1.76 × 10^−27^	1.48 × 10^−21^
B*44	7 (6.2%)	0 (0.0%)	20.1 ( 2.6–157.5)	0.003	> 0.05
B*1301 or B*44	89 (78.8%)	13 (9.2%)	36.8 (17.8–76.1)	1.77 × 10^−31^	1.49 × 10^−25^

*p*_c_ Values and ORs were calculated as described in “Materials and Methods.”
